# Efficacy and safety of Tripterygium wilfordii Hook F preparations for the treatment of Crohn disease: A systemic review and meta-analysis protocol

**DOI:** 10.1097/MD.0000000000016231

**Published:** 2019-06-28

**Authors:** Jianying Zhang, Gang Han, Zhenwei Yu

**Affiliations:** Sir Run Run Shaw Hospital, School of Medicine, Zhejiang University, Hangzhou, China.

**Keywords:** complementary medicine, Crohn disease, traditional Chinese medicine, Tripterygium wilfordii Hook F

## Abstract

**Background:**

Crohn disease (CD) is associated with substantial healthcare related costs and impairment of quality of life. Tripterygium wilfordii Hook F (TwHF) is proved to be effective for CD in animal and human. However, there is no systemic review and meta-analysis regarding the clinical efficacy and safety of TwHF preparation for the treatment of CD.

**Methods:**

Six electronic databases (Medline, EMBASE, Cochrane database, Chinese National Knowledge Infrastructure, Wanfang Database and Chongqing VIP Database) will be searched for eligibility studies. Data from the included studies will be extracted and the quality of studies will be assessed. Data synthesis will be performed using Review Manager software. Sensitivity analysis and publication bias assessment will also be carried out.

**Results:**

This systemic review and meta-analysis will provide synthesized result of clinical efficacy and safety of TwHF preparation for the treatment of CD.

**Conclusion:**

This research will determine the clinical efficacy and safety of TwHF preparation for the treatment of CD.

Registration: PROSPERO CRD42019127893

## 1 Introduction

Crohn disease (CD) is a chronic inflammatory disease of the gastrointestinal tract and can cause bowel damage and disability. ^[1]^ It has become a global disease with accelerating incidence in newly industrialized countries, including China.^[2,3]^ Although incidence in western countries is stabilizing, burden remains high.^[3]^ Data from a variety of studies indicates that CD is associated with substantial direct and in-direct costs across patients’ lifespan.^[4,5]^ Symptoms of active CD, such as diarrhea and abdominal pain, have been shown to impact health related quality of life substantially in large multinational studies.^[6,7]^

The exact mechanism of CD remains to be elucidated.^[8]^ The aim of current treatment for CD is preventing complications and halting the progressive course of disease.^[1]^ There are many drugs can be selected for the treatment based on the severity of CD, such as corticosteroids, mesalamine, sulfasalazine, budesonide and so on.^[9,10]^ Biological therapies including anti-tumor necrosis factor-α antibodies and anti-integrin antibodies have been introduced into the treatment of CD in the recent decades and achieved good clinical response rate.^[11]^ However, the current therapeutic effect is not satisfied. Hospitalization and surgery are common in these patients. One-fifth of patients with CD experienced intestinal complications and half of patients required hospitalization within the first year after diagnosis. And surgery occurred in half of patients within 10 years after diagnosis.^[12]^

Natural products and herbal medicine have exhibited efficacy in preclinical and clinical evaluation, improved symptoms, and decreased medical costs for CD patients.^[13,14]^ Extracts of Tripterygium wilfordii Hook F (TwHF) have been used to treat inflammatory and autoimmune disease in China for many years.^[15]^ The main active components of TwHF exhibit good effect on CD in animal models through different mechanisms.^[16,17]^ Some clinical studies had shown that TwHF may be both effective and safe for patients with CD.^[18,19]^ However, the clinical evidence of TwHF for the treatment of CD has not been well concluded. Here we describe a proposed systemic review and meta-analysis protocol to evaluate the clinical efficacy and safety of TwHF preparations for the treatment of CD.

## 2 Methods

This protocol is written following the Preferred Reporting Items for Systemic Review and Meta Analysis Protocol (PRISMA-P) statement.^[20]^ And this protocol is registered in PROSPERO (CRD42019127893). Ethical approval is not needed because this research only involves published data.

### 2.1 Eligibility criteria

#### 2.1.1 Type of study

Only randomized trials (RCTs) will be included. There is no restriction in masking or blinding.

#### 2.1.2 Participants

Participants with a diagnosis of CD will be included. There is no restriction regarding the diagnostic criteria used in the original study. However, patients aged below 18 will be excluded. Studies which enroll patients less than 20 will be excluded.

#### 2.1.3 Interventions

Patients treated with TwHF preparation alone or combined with other therapeutics. TwHF preparation is defined as drugs containing TwHF extracts or active components of TwHF. Traditional formulations containing TwHF, such as decoction, haustus and mixture, are also considered as TwHF preparations. There is no restriction regarding the administered does or frequency.

#### 2.1.4 Comparison

Patients treated with any drug other than TwHF preparations. Patients not treated or treated with placebo will also be included.

#### 2.1.5 Language

There is no restriction regarding reporting language.

### 2.2 Information source

The following electronic databases will be searched: Medline, EMBASE, Cochrane database, Chinese National Knowledge Infrastructure (CNKI), Wanfang Database and Chongqing VIP Database.

### 2.3 Search strategy

The electronic databases will be searched using a combination of following items: Tripterygium wilfordii Hook F; TwHF; Tripterygium; Triptergium; triptolide; triptergium glycoside; Lei Gong Teng; Thunder God vine; Crohn(s) disease. Search strategy will be adjusted to suit the specific database. Relevant studies, such references of included studies, will be searched manually. Studies published before Apr 1st, 2019 will be sought. The literature search will be carried out by 2 reviewers independently (JYZ and GH). The search will be done again before data synthesis.

### 2.4 Study records

#### 2.4.1 Study selection

The searched studies will be managed using a reference managing software, NoteExpress (ANGEAN Technology, Beijing, China). Two reviewers (JYZ and GH) will review and select studies according to eligibility criteria independently. Any discrepancies will be discussed and solved with a third reviewer (ZWY).

#### 2.4.2 Data collection

The extracted data will be managed using an Excel electronic table. The following items will be collected from the included studies: first author, published year, study duration and religion, number, sex and age of participants, severity of illness, intervention of experimental group and control group, outcomes, and adverse events. If required is not presented in the published study, the reviewer (ZWY) will contact the original authors for missing data. Study will be excluded if main outcome data cannot be obtained. The data will be collected by 2 reviews (JYZ and GH) independently, and any discrepancies will be solved with a third reviewer (ZWY).

### 2.5 Outcomes

#### 2.5.1 Main outcome

Clinical effective rate, Clinical response, remission, or fistula healing defined by the original study is recognized as clinical effective.

#### 2.5.2 Secondary outcomes

(1) Adverse event rate. (2) Other measurements of disease activity (such as endoscopic remission, clinical recurrence).

### 2.6 Risk of bias in individual studies

Cochrane risk of bias tool will be used for the evaluation of risk of bias in individual studies.^[21]^ The following 7 items will be investigated separately: random sequence generation, allocation concealment, blinding, incomplete outcome data, selective reporting bias, and other bias. Each item will be evaluated as “Low risk”, “High risk” or “Unclear”. And each included study will also be evaluated as “Low risk”, “High risk” or “Unclear” based on the results of the 7 items. Two reviewers (JYZ and GH) will evaluate the risk of bias independently and any discrepancies will be solved with a third reviewer (ZWY).

### 2.7 Date synthesis

The data will be reviewed before synthesis. If included studies are not enough, the data will be qualitatively represented. Data synthesis will be carried out using Review Manage software (version 5.3, Copenhagen: the Nordic Cochrane center, The Cochrane collaboration 2014). Categorical outcomes will be synthesized using OR value and 95% confidential interval (CI). Mean difference (MD) and 95% CI will be calculated for continuous results. Heterogeneity among included studies will be evaluated using I^2^ test. I^2^ > 50% is defined as significant heterogeneity exists among studies. Mantel-Haenszel fixed effect model will be used for data synthesis if no heterogeneity exists; or a random effect model will be used.

Subgroup analysis will be carried out based on following items if included studies are sufficient:

(1) types and doses of TwHF preparations;
(2) type of combined therapies;
(3) severity of illness.

Sensitivity analysis will be carried out using leave-one-out method. Briefly, the main outcome will be re-synthesized by excluding studies one by one and the robustness of results will be evaluated. Publication bias will be investigated using funnel plots if included studies are more than 10.

### 2.8 Summary

The results will be summarized using the Grading of Recommendations Assessment, Development, and Evaluation approach.^[22]^

## 3 Discussion

To the best of our knowledge, this research will be the first systemic review and meta-analysis of RCTs evaluating the clinical efficacy and safety of TwHF for the treatment of CD. This research will provide valuable information for clinicians with respect to treating CD. We believe that complementary medicine, such as TwHF, is still important for the treatment of disease. Further pharmacoeconomic studies evaluating the cost-utility of TwHF for the treatment of CD is required. However, the major limitation will be the quality of included studies, which will affect the credibility of this systemic review and meta-analysis.

## Author contributions

ZWY had the original idea for a systemic review and meta-analysis. JYZ and GH designed the protocol. JYZ acquired funding. JYZ and ZWY reviewed the search strategy. JYZ and GH drafted the protocol. ZWY registered the protocol in PROSPERO. ZWY is the guarantor of the protocol. All authors read and approved the final version.

**Conceptualization:** Zhenwei Yu.

**Funding acquisition:** Jianying Zhang.

**Methodology:** Jianying Zhang, Gang Han.

**Validation:** Zhenwei YU.

**Writing – original draft:** Jianying Zhang, Gang Han.

**Writing – review & editing:** Jianying Zhang, Gang Han, Zhenwei YU.

Zhenwei YU orcid: 0000-0002-3776-2290.
